# A Homogeneous Polysaccharide from Fructus *Schisandra chinensis* (Turz.) Baill Induces Mitochondrial Apoptosis through the Hsp90/AKT Signalling Pathway in HepG2 Cells

**DOI:** 10.3390/ijms17071015

**Published:** 2016-06-28

**Authors:** Yonglin Chen, Songshan Shi, Huijun Wang, Ning Li, Juan Su, Guixin Chou, Shunchun Wang

**Affiliations:** 1 The MOE Key Laboratory for Standardization of Chinese Medicines, Shanghai University of Traditional Chinese Medicine, Shanghai 201203, China; syxcyl@foxmail.com (Y.C.); shisongshan1978@126.com (S.S.); wanghuijun666666@163.com (H.W.); miss1991lining@163.com (N.L.); ssujuan77@163.com (J.S.); 2 The SATCM Key Laboratory for New Resources and Quality Evaluation of Chinese Medicines, Shanghai University of Traditional Chinese Medicine, Shanghai 201203, China; 3 Institute of Chinese Materia Medica, Shanghai University of Traditional Chinese Medicine, Shanghai 201203, China

**Keywords:** hepatocellular carcinoma, *Schisandra chinensis*, polysaccharide, mitochondrial apoptosis, autophagy, Hsp90/AKT signalling pathway

## Abstract

According to the potential anti-hepatoma therapeutic effect of *Schisandra chinensis* polysaccharides presented in previous studies, a bioactive constituent, homogeneous *Schisandra chinensis* polysaccharide-0-1 (SCP-0-1), molecular weight (*M*_W_) circa 69.980 kDa, was isolated and purified. We assessed the efficacy of SCP-0-1 against human hepatocellular liver carcinoma (HepG2) cells to investigate the effects of its antitumour activity and molecular mechanisms. Anticancer activity was evaluated using microscopy, 3-[4,5-dimethyl-2-thiazolyl]-2,5-diphenyltetrazolium bromide (MTT) assay, Hoechst 33258 staining, acridine orange (AO) staining, flow cytometry (FCM), and cell-cycle analysis. SCP-0-1 inhibited the HepG2 cells’ growth via inducing apoptosis and second gap/mitosis (G2/M) arrest dose-dependently, with a half maximal inhibitory concentration (IC_50_) value of 479.63 µg/mL. Western blotting of key proteins revealed the apoptotic and autophagic potential of SCP-0-1. Besides, SCP-0-1 upregulated Bcl-2 Associated X Protein (Bax) and downregulated B-cell leukemia/lymphoma 2 (Bcl-2) in the HepG2 cells. The expression of caspase-3, -8, and -9; poly (ADP-ribose) polymerase (PARP); cytochrome c (Cyt C); tumor protein 53 (p53); survivin; sequestosome 1 (p62); microtubule-associated protein 1 light chain-3B (LC3B); mitogen-activated protein kinase p38 (p38); extracellular regulated protein kinases (ERK); c-Jun N-terminal kinase (JNK); protein kinase B (AKT); and heat shock protein 90 (Hsp90) were evaluated using Western blotting. Our findings demonstrate a novel mechanism through which SCP-0-1 exerts its antiproliferative activity and induces mitochondrial apoptosis rather than autophagy. The induction of mitochondrial apoptosis was attributed to the inhibition of the Hsp90/AKT signalling pathway in an extracellular signal-regulated kinase-independent manner. The results also provide initial evidence on a molecular basis that SCP-0-1 can be used as an anti-hepatocellular carcinoma therapeutic agent in the future.

## 1. Introduction

Liver cancer, also called hepatocellular carcinoma (HCC), is one of the most frequently presented carcinoma, and the third-greatest cause of lethal malignancy tumor worldwide. The mortality rate of HCC is increasing, and approximately 85% of HCC cases occur in developing countries [1]. At present, the incidence of HCC is second in China, the number of incident cases and deaths continue to increase year by year [2]. HCC is the most common cancer in people aged younger than 60 years and the leading cause of cancer death in Chinese men [3]. Most HCC patients are diagnosed as terminal. Although chemotherapy is a common therapeutic strategy after surgery, its application is limited due to its serious adverse effects and growing multidrug resistance in tumour cells. Therefore, finding new nontoxic anticancer drugs with fewer side effects is imperative [4]. Some phytochemicals that possess anticancer properties such as suppressing cell proliferation and inducing cell death have recently received considerable attention [5].

Fructus *Schisandra chinensis* (Turz.) Baill is a well-known traditional Chinese medicine and health food commonly called WuWeiZi or ‘five-flavour fruit’ in China. Northeast Asia is the main growing area, ranging from Northeast China, the Russian Far East, and the Korean Peninsula to Japan. In China, *S. chinensis* is officially listed in the Chinese Pharmacopoeia as a tonic and used extensively for treating chronic or acute diseases including viral or chemical hepatitis, high cholesterol, hypertension, hyperglycaemia, and atherosclerosis [6]. In addition, *S. chinensis* supresses cell proliferation and induces apoptosis in several cancer cell lines including U251 and U87 glioma cells [7], human epithelial colorectal adenocarcinoma Caco-2 cells [8], U937 leukaemia cells [9], colon adenocarcinoma LoVo and ovarian adenocarcinoma 2008 cells [10], human colon cancer HCT116c cells [11], human colorectal carcinoma HT-29 cells [12], mouse melanoma B16F0 cells [13], and mouse breast cancer 4T1 cells [14]. Several reports have suggested that the major bioactive constituents of *S. chinensis* are the dibenzocyclooctadiene lignans schisandrin A, schisandrin B [15], gomisin A, gomisin N [16], essential oils [17], and polysaccharides [18].

In recent years, studies have shown that polysaccharides from *S. chinensis* exert biological effects on cells and exhibit antitumour activity against renal cell carcinoma Caki-1 cells [18,19], Heps-bearing mice [20], and human hepatocellular liver carcinoma (HepG2) cells [21]. Moreover, *S. chinensis* polysaccharides combined with 5-fluorouracil (5-Fu) exhibit enhanced antitumour activity and a low molecular weight purified polysaccharide from *S. chinensis* (SCPP11) can decrease the 5-Fu-induced toxicity effect [20]. However, the effect of the antitumour activity of homogeneous polysaccharides isolated from *S. chinensis* on the growth of HepG2 cells has not been investigated. Furthermore, whether *S. chinensis* polysaccharide-0-1 (SCP-0-1) can induce apoptosis and autophagy and their molecular mechanisms remains unclear. Therefore, we tested the apoptotic and autophagic potential of a novel homogeneous polysaccharide, SCP-0-1, isolated from *S. chinensis*, in HepG2 cells.

To explore the anticancer activity of SCP-0-1 and its mechanisms in regulating cellular processes of cancer cell, this study examined the effect on proliferation, distribution of cell-cycle, apoptosis, as well as autophagy in hepatoma HepG2 cells. The apoptosis induction was confirmed by a great deal of experiments: microscopy, 3-[4,5-dimethyl-2-thiazolyl]-2,5-diphenyltetrazolium bromide (MTT) assay, Annexin-V/phosphatidylinositol (PI) staining, Hoechst 33258 staining, acridine orange (AO) staining, cell-cycle analysis, and the immunoexpression of key apoptotic and autophagic proteins. SCP-0-1 engendered apoptosis through mitochondrial apoptosis via blocking cell cycle at the period of second gap/mitosis (G2/M) phase and inducing cell apoptosis; enhancing the cleavage of caspase-9, caspase-3, and poly (ADP-ribose) polymerase (PARP); upregulating the Bcl-2 Associated X Protein/B-cell leukemia/lymphoma 2 (Bax/Bcl-2) ratio; and translocating cytochrome c (Cyt C). Moreover, SCP-0-1 induced tumor protein 53 (p53)-dependent apoptosis in HepG2 cells. This is the first evidence that SCP-0-1 induces mitochondrial apoptosis rather than autophagy by inhibiting the heat shock protein 90 (Hsp90)/protein kinase B (AKT) signalling pathway in HepG2 cells.

## 2. Results

### 2.1. Extraction, Separation and Purification of S. chinensis Polysaccharide-0-1

**Figure 1:**
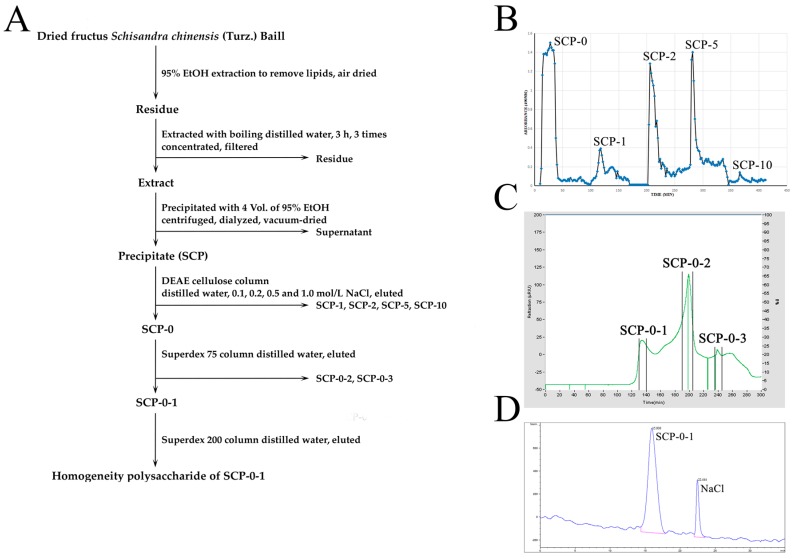
Flow diagram of isolation and purification of homogeneous *S. Chinensis* polysaccharide (SCP-0-1) from fructus *S. chinensis* (Turz.) Baill. (**A**) The dried fructus *S. chinensis* were defatted with EtOH, and extracts were obtained by boiling in distilled water; the extract was obtained and then precipitated to gain fructus *S. chinensis*. After continuous separation via diethylaminoethyl (DEAE)-cellulose anion-exchange chromatography and Superdex 75 and 200 gel permeation chromatography, the carbohydrate fraction SCP-0-1 was obtained; (**B**) The eluted curve of polysaccharides from *S. Chinensis* polysaccharides (SCP) was fractionated on a DEAE-cellulose column (50 × 5 cm, Cl^−^ form) and eluted with distilled water and different gradients of NaCl at 1.0 mL/min; (**C**) The eluted curve of SCP-0 fractionated on a Superdex 75 column (SCP-0-1: 130–140 min, SCP-0-2: 190–205 min, and SCP-0-3: 235–245 min); (**D**) The eluted curve of SCP-0-1 in HPGPC. The sample was analysed by a Shodex series-connected KS-804 and KS-802 gel filtration column (30 cm × 7.8 mm) and eluted with 0.2 mol/L NaCl at 0.8 mL/min.

*S. chinensis* polysaccharide-0-1 (SCP-0-1) was obtained as depicted in the flow diagram in Figure 1A. The dried fructus *S. chinensis* (Turz.) Baill (10 kg) were defatted with 95% EtOH and then boiled in distilled water to obtain the extract of the defatted fruits. Then we precipitated the extract with 4 volumes of 95% EtOH to obtain the crude polysaccharide *S. chinensis* polysaccharides (SCP) (1.206 kg, yield: 12.06%). After continuous separation by using diethylaminoethyl (DEAE)-cellulose anion-exchange chromatography and Superdex 75 and 200 gel permeation chromatography (Figure 1B,C), 0.958% of the crude polysaccharide was obtained as a homogeneous polysaccharide, SCP-0-1, which was confirmed using high-performance gel permeation chromatography (HPGPC) (Figure 1D); the molecular weight of SCP-0-1 was estimated to be 69.980 kDa in reference to P-series Dextran (Figure S1).

### 2.2. Antiproliferative Activity of S. chinensis Polysaccharide-0-1

**Figure 2:**
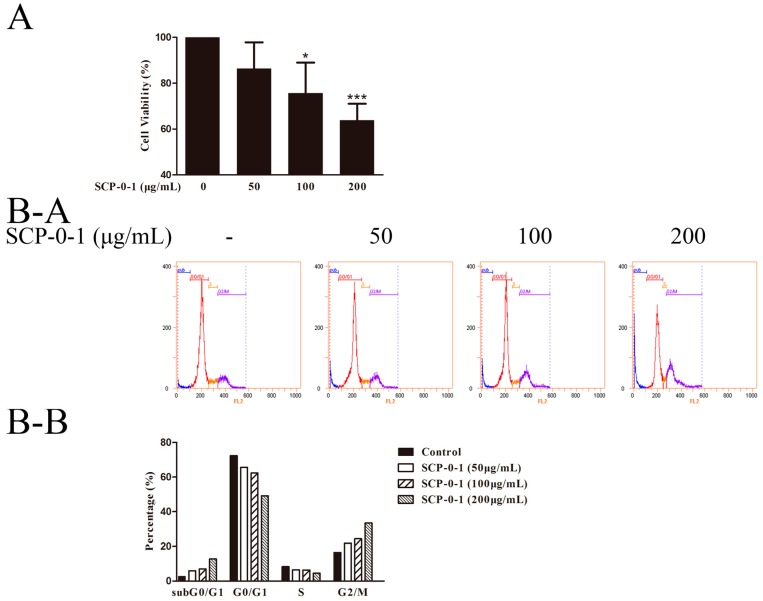

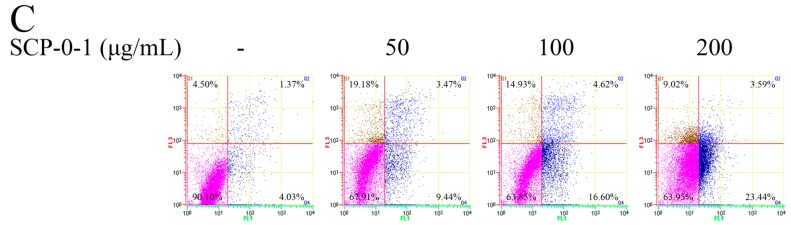
The effectiveness of S. Chinensis polysaccharide-0-1 (SCP-0-1) on cell viability and apoptosis in human hepatocellular liver carcinoma (HepG2) cells. (**A**) The cells were incubated with SCP-0-1 (0–200 µg/mL) for 24 h as determined via adopting the 3-[4,5-dimethyl-2-thiazolyl]-2,5-diphenyltetrazolium bromide (MTT) assay. Its related data were presented as the mean values ± SD of three independent experiments. * *p* < 0.05 and *** *p* < 0.001 versus control group; (**B**) Effect of SCP-0-1 on the cell-cycle and sub zero gap/first gap (subG0/G1) apoptotic population of the HepG2 cells. The HepG2 cells were treated with SCP-0-1 (0–200 µg/mL) and fixed for 24 h for flow cytometry (FCM). The deoxyribonucleic acid (DNA) content of propidium iodide-labelled nuclei was analysed. The populations of subG0/G1 apoptosis and of zero gap/first gap (G0/G1), synthesis (S), and second gap/mitosis (G2/M) phase were quantified by using DNA histograms; (**C**) Cell death of cells assessed using flow cytometry. Cell apoptosis detection with flow cytometry are proceeded by adopting the Annexin-V/phosphatidylinositol (PI) Apoptosis Detection Kit.

To judge the effect of SCP-0-1 on hepatoma cell proliferation, we plated the human hepatocellular liver carcinoma (HepG2) cells into 96-well microtiter plates for 24 h (5 × 10^3^ cells per well) and subsequently treated them with fresh medium SCP-0-1 (0-200 μg/ml). As shown in Figure 2A, SCP-0-1 exhibited antiproliferative activity against the hepatoma HepG2 cells dose-dependently, and the half maximal inhibitory concentration (IC_50_) value of SCP-0-1 for the tumour cells was 479.63 µg/mL.

SCP-0-1 significantly inhibited the proliferation of the HepG2 cells compared with the control group. Also it increased or reduced the proportion of HepG2 cells in various cell-cycle phases: The percentage in the zero gap/first gap (G0/G1) and synthesis (S) phases was reduced, while that in the second gap/mitosis (G2/M) phase was significantly increased. The G2/M phase rate in the four groups of samples were 16.52%, 21.92%, 24.36%, and 33.48%, respectively (Figure 2B). Flow cytograms showed a much higher percentage of the SCP-0-1-treated cells in sub zero gap/first gap (subG0/G1) (12.78%; Figure 2B-A and Figure 2B-B) than that of the control cells (2.79%; Figure 2B-A and Figure 2B-B). Regarding cell-cycle arrest, SCP-0-1 exerted its antitumour activity through blocking cell cycle at the period of G2/M phase and preventing cell mitosis in HepG2 cells.

In order to study whether the reduced cell proliferation was due to SCP-0-1-elicited apoptosis, we determined the apoptotic population and cell death in the SCP-0-1-treated HepG2 cells by adopting Annexin-V/phosphatidylinositol (PI) staining and flow cytometry (FCM), Q1, Q2, Q3, and Q4 denote necrotic, late apoptotic, viable (live), and early apoptotic regions, respectively. The proportion of apoptotic cells increased from 5.40% to 27.03% dose-dependently (Figure 2C), indicated that SCP-0-1 induces the apoptosis of HepG2 cells.

### 2.3. Cell Morphology Demonstrated that S. chinensis Polysaccharide-0-1 Induced Apoptosis Rather than Autophagy

**Figure 3:**
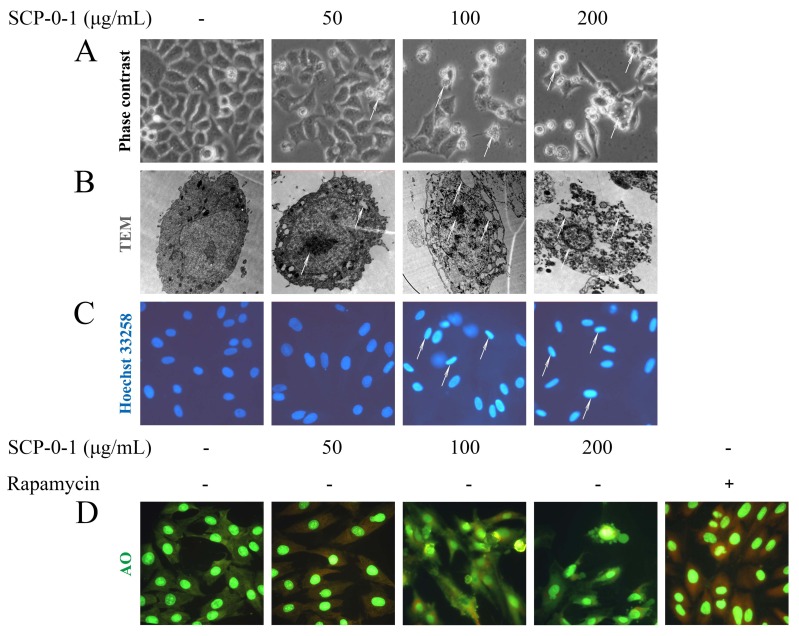
Morphological changes in human hepatocellular liver carcinoma (HepG2) cells after *S. Chinensis* polysaccharide-0-1 (SCP-0-1) treatment. The morphological observation confirmed that SCP-0-1 induced evident apoptosis rather than autophagy in the HepG2 cells. (**A**,**B**) Morphological changes induced by SCP-0-1. The cells were incubated with SCP-0-1 (0-200 µg/mL) for 24 h. After treatment, cellular morphology was observed under a phase contrast microscope (magnification: 400×) and transmission electron microscope (magnification: 4200×); An inhibitory effect of SCP-0-1 on cell growth is observed as being accompanied by membrane blebbing, arrows denote membrane blebbing and condensed chromatin, which are characteristic of apoptosis; (**C**) Nuclear morphology staining using Hoechst 33258; the morphological characterisation of cell nuclei was analysed under a fluorescence microscope (magnification: 400×). Condensed chromatin are shown to contain compacted chromatin; arrows point to condensed chromatin which is characteristic of apoptosis; (**D**) SCP-0-1 did not induce autophagy in the HepG2 cells. Autophagy was detected through acridine orange staining. Cells were sowed in a six-well plate, treated with the specified concentrations of SCP-0-1 or 10 nmol/L rapamycin for 24 h. The cells were observed under the fluorescence microscope (magnification: 400×).

The inhibitory effect of SCP-0-1 on cell growth was accompanied by membrane blebbing, as observed using a phase contrast microscope (Figure 3A), indicating that cell death was due to apoptosis death. Ultrastructural morphological changes were observed by transmission electron microscope (TEM) in the subcellular level. Typical apoptotic features include a range of morphological changes like cell membrane blebbing, cytoplasmic shrinkage, and nuclear chromatin condensation or cleavage [22]. As shown in Figure 3B and Figure S3, the control cells display normal cell morphology including intact cell membranes and normal nuclei. In contrast, after treatment with 100–200 µg/mL SCP-0-1 for 24 h, TEM showed that cell shrinkage, cytoplasmic vacuolization, and condensation of nuclear chromatin could be observed, and finally, that typical apoptosis bodies were formed. In addition, TEM was considered the most general approach for monitoring autophagy since it could reveal the existence of autophagosomes in cells. As shown in Figure 3B, the ultrastructural analysis by TEM further verified the inexistence of autophagosomes with double membrane in SCP-0-1-treated HepG2 cells. No ultrastructural evidence of autophagy was observed. Altogether, these data collectively demonstrate that SCP-0-1 induces HepG2 cells apoptosis rather than autophagy.

We examined the morphological changes in response to SCP-0-1 treatment by performing Hoechst 33258 staining. Consequently, the control cells exhibited normal cell morphology with round and homogeneous nuclei. By contrast, the cells treated with SCP-0-1 presented the morphological characteristics of apoptotic cells, with clearly observable typical condensed chromatin and fragmented nuclei (Figure 3C and Figure S2).

The induction of autophagy ranges from an increased formation of autophagosomes, to a sequestration of cytoplasmic proteins and organelles into the lysosomal component, and is characterised by the formation of acidic vesicular organelles [23]. To test the autophagic response of the HepG2 cells after SCP-0-1 treatment, we analysed acidic vesicular organelles (AVO) formation through acridine orange (AO) staining. The photo showed that few or no AVO were observed; after rapamycin treatment, there are some AVO in cells (Figure 3D) with amendments highlighted in red. The results of AO staining showed that rapamycin induced AVO formation in HepG2 cells, while the number of AVO was negligible SCP-0-1-treated cells, proved that SCP-0-1 did not affect the autophagy of the HepG2 cells.

### 2.4. Changes in Proteins Expression Involved in Apoptosis and Autophagy

**Figure 4:**
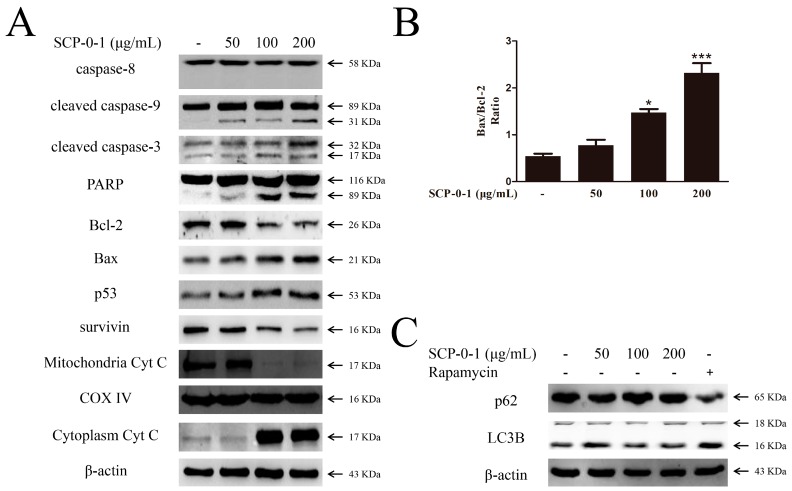
*S. Chinensis* polysaccharide-0-1 (SCP-0-1) activated apoptosis signalling rather than autophagy signalling in human hepatocellular liver carcinoma (HepG2) cells. (**A**) The cells were incubated with SCP-0-1 (0–200 µg/mL) for 24 h then harvested and immunoblotted with antibodies against the specified proteins; β-actin being a loading control was used for cytoplasmic and whole-cell extracts, cytochrome c oxidase IV (COX IV) being a loading control was used for mitochondrial extracts; (**B**) Effect of SCP-0-1 on proapoptotic Bcl-2 Associated X Protein (Bax) and antiapoptotic B-cell leukemia/lymphoma 2 (Bcl-2) levels in HepG2 cells. The Bax/Bcl-2 ratio in cells treated with the specified concentrations of SCP-0-1. Its related data were presented as the mean values ± SD of three independent experiments. * *p* < 0.05 and *** *p* < 0.001 *versus* control group; (**C**) Effect of specified concentrations of SCP-0-1 or 10 nmol/L rapamycin on the expression status of specific autophagic markers, sequestosome 1 (p62) and microtubule-associated protein 1 light chain-3B (LC3B).

Caspase-3 causes proteolytic cleavage of some key proteins like the nuclear enzyme poly (ADP-ribose) polymerase (PARP), so it is a critical executioner of apoptosis [24]. We observed that SCP-0-1 upregulated cleaved caspase-3 levels and evoked the cleavage of PARP in the HepG2 cells (Figure 4A).

To investigate whether SCP-0-1 induces the apoptosis of HepG2 cells associated with intrinsic mitochondrial apoptosis pathway, we measured the levels of B-cell leukemia/lymphoma 2 (Bcl-2) family members (Bcl-2 Associated X Protein (Bax) and Bcl-2). Results show that the Bax/Bcl-2 ratios in the four sample groups were 0.54, 0.78, 1.47, and 2.32, respectively, indicating that SCP-0-1 treatment reduced Bcl-2 levels and increased Bax levels (Figure 4A,B).

Since Bcl-2 had a cooperative relationship with survivin in inhibiting cell apoptosis [25], we evaluated the survivin and Bcl-2 levels in the HepG2 cells. The result showed that the survivin levels decreased (Figure 4A).

Conversion of microtubule-associated protein 1 light chain-3A (LC3A) to microtubule-associated protein 1 light chain-3B (LC3B) is usually a marker of autophagosome formation, and sequestosome 1 (p62) is packed into an autophagosome and decomposed in the autolysosome afterwards [26]. We investigated microtubule-associated protein 1 light chain-3 (LC3) conversion and p62 expression by using Western blotting; the results revealed that, compared with rapamycin, SCP-0-1 failed to regulate the LC3B levels and that p62 expression did not significantly change in response to SCP-0-1 in the HepG2 cells, as shown in Figure 4C. These results implied that SCP-0-1 did not affect autophagy.

### 2.5. Changes in Protein Expression Involved in Mitogen-Activated Protein Kinase/Phosphoinositide 3-Kinase/Heat Shock Protein 90 Signalling Pathways

**Figure 5:**
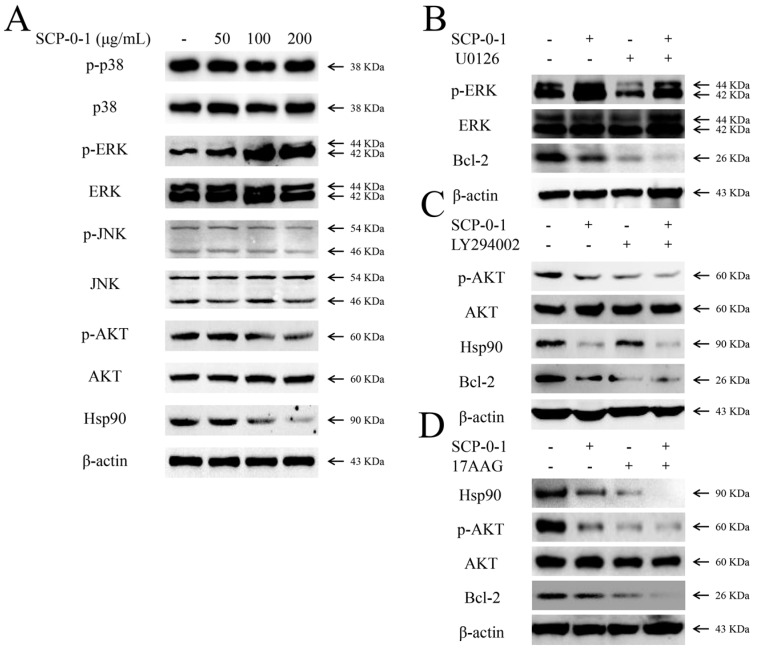
Effects of *S. Chinensis* polysaccharide-0-1 (SCP-0-1) on the mitogen-activated protein kinase (MAPK), phosphatidylinositol 3-kinase (PI3K)/protein kinase B (AKT), and heat shock protein 90 (Hsp90) signalling pathways in human hepatocellular liver carcinoma (HepG2) cells. (**A**) After treatment with 100–200 μg/mL SCP-0-1 for 24 h, and the expression of phosphorylated p38 mitogen-activated protein kinase (p-p38), mitogen-activated protein kinase p38 (p38), phosphorylated extracellular signal-regulated protein (p-ERK), extracellular regulated protein kinases (ERK), phosphorylated c-Jun N-terminal kinase (p-JNK), c-Jun N-terminal kinase (JNK), phosphorylated protein kinase B (p-AKT), protein kinase B (AKT), and Hsp90 was measured using immunoblotting. Inhibition of p-ERK, p-AKT, and Hsp90 enhanced SCP-0-1-triggered cell death in HepG2 cells; (**B**) Immunoblot analyses for p-ERK, ERK, and B-cell leukemia/lymphoma 2 (Bcl-2) by treatment with the ERK inhibitor 1,4-Diamino-2,3-dicyano-1,4-bis(2-aminophenylthio)-butadiene (U0126) (12 µmol/L). After treatment with 100–200 μg/mL SCP-0-1 for 24 h in the with or without of the ERK inhibitor U0126; (**C**,**D**) The cells were preconditioned by the phosphatidylinositol 3-kinase (PI3K) inhibitor 2-(4-morpholino)-8-phenyl-4H-1-benzopyran-4-one (LY294002) (60 µmol/L) and Hsp90 inhibitor 17-Allylamino-17-demethoxygeldanamycin (17-AAG) (120 nmol/L) for 1 h, After treatment with 100–200 μg/mL SCP-0-1 for 24 h, the cells were collected and immunoblotted with antibodies against the indicated proteins.

Studies have extensively documented that the mitogen-activated protein kinase (MAPK) signalling pathways play a critical role in modulating apoptosis and autophagy [27,28]. In the current study, the effects of SCP-0-1 on this pathway have been investigated. SCP-0-1 treatment increased the phosphorylation of extracellular regulated protein kinases (ERK) (Figure 5A). However, SCP-0-1 had no appreciable effects on phosphorylated p38 mitogen-activated protein kinase (p-p38) and phosphorylated c-Jun N-terminal kinase (p-JNK).

The phosphoinositide 3-kinase (PI3K)/protein kinase B (AKT)/mechanistic target of the rapamycin (mTOR) signalling pathway is one of the major pathways regulating autophagy [29]. As shown in Figure 5A, SCP-0-1 treatment reduced the AKT phosphorylation concentration-dependently. However, the LC3B and p62 levels showed almost no change (Figure 4C), suggesting that SCP-0-1 does not regulate autophagy through the PI3K/AKT/mTOR signalling pathway.

Heat shock protein 90 (Hsp90), a highly conserved molecular chaperone, is involved in numerous crucial cellular processes, including proliferation, migration, and angiogenesis, through the stabilisation of its client proteins [30]. We examined the protein expression of Hsp90. The results showed that Hsp90 expression concentration-dependently decreased in response to SCP-0-1 (Figure 5A).

### 2.6. Inhibition by S. chinensis Polysaccharide-0-1 Is Attributed to the Repression of the Heat Shock Protein 90/Protein Kinase B Signalling Pathway

As shown in Figure 5A, SCP-0-1 (>100 µg/mL) reduced the AKT phosphorylation and protein expression of Hsp90 and increased phosphorylated extracellular signal-regulated protein (p-ERK) expression in a dose-dependent manner. Therefore, to trace out which pathway is involved in the cytotoxicity of SCP-0-1, we evaluated the actions of a group of specific inhibitors, containing 1,4-Diamino-2,3-dicyano-1,4-bis(2-aminophenylthio)-butadiene (U0126), 2-(4-morpholino)-8-phenyl-4H-1-benzopyran-4-one (LY294002), and 17-Allylamino-17-demethoxygeldanamycin (17-AAG), on SCP-0-1-induced cell death.

The inhibition of ERK activation with ERK inhibitor (U0126) abolishes Bcl-2 activation. In this study, the SCP-0-1-caused downregulation levels of Bcl-2 and SCP-0-1-induced apoptosis was not abolished by the pretreatment of ERK inhibitor (U0126) in HepG2 cells (Figure 5B). These results suggesting that ERK activation may not directly participate in SCP-0-1-induced apoptosis of HepG2 cells.

Pretreatment of cells with LY294002 (a PI3K inhibitor) and 17-AAG (an Hsp90 inhibitor) respectively suppressed the protein expression of phosphorylated protein kinase B (p-AKT) and Hsp90. These inhibitors also promoted cell death and further reduced the SCP-0-1-mediated downregulation of Bcl-2 (Figure 5C,D), demonstrating that SCP-0-1 partly induced apoptosis through the Hsp90/AKT signalling pathway.

## 3. Discussion

More and more studies have shown the potential therapeutic effect of *S. Chinensis* against cancer [2,3,4,5,6,7,8,9,10,11,12,13,14,18,19,20,21]. However, the underlying mechanisms have yet to be explained. Prior studies have shown that *S. chinensis* polysaccharides might be a promising new antihepatoma agent according to the following observations. First, the potential mechanisms underlying SCP-induced apoptosis and its antiangiogenic activity might have association with the increase of Bax and tumor protein 53 (p53), decrease of Bcl-2, and reduction of the vascular endothelial growth factor (VEGF), platelet endothelial cell adhesion molecule-1 (CD31), and cell adhesion molecule CD34 in xenografted tumours [19]; Second, an in vivo study in 5-fluorouracil (5-Fu)-treated Heps-bearing mice proved that a low molecular weight purified polysaccharide from *S. chinensis* (SCPP11) combined with 5-Fu exhibits elevated antitumour activity; moreover, SCPP11 could weaken the 5-Fu-induced toxicity effectiveness [20]; Third, through protecting the immune system, SCP could availably avoid immune injury during radiotherapy, and it wouldn’t cause causing any side effects in normal mice [31]. In the current study, SCP-0-1 was isolated from SCP-0 (Figure 1), and its inhibitory effect on human hepatocellular liver carcinoma (HepG2) cell growth was evaluated. This is the earliest report disclosing the bioactivity and molecular events of SCP-0-1 in HepG2 cells.

In our study, we tested the antiproliferative activity of SCP-0-1 and its mechanisms of action for HepG2 cells. We determined that SCP-0-1 dose-dependently suppressed the growth of the HepG2 cells. Moreover, SCP-0-1 treatment induced G2/M arrest in the HepG2 cells. The ratio of G2/M phase was increased, and that in the subG0/G1 phase was significantly increased concentration-dependently. SCP-0-1 dose-dependently increased the number of apoptotic cells, as assessed using FCM (Figure 2). These results demonstrate that the SCP-0-1-induced growth inhibition is related to the arrest of the HepG2 cells in the G2/M phase and subG0/G1 phase, like the action of paclitaxel, as an earlier study disclosed [32].

Treatment with 50 µg/mL SCP-0-1 yielded an apoptotic index of 7.51%; we hypothesised that autophagy may be trapped in the inhibition of cell proliferation and cell death. Autophagy might be a tactic to avoid cell apoptosis at the beginning of the medication process. Specifically, under certain conditions, autophagy protects the cell from death by adapting certain mechanisms, thus inhibiting apoptosis. This hypothesis was confirmed by additional experiments (Figure 2C). In conclusion, our study demonstrates that 50 µg/mL SCP-0-1 triggers protective autophagy and limited apoptosis in HepG2 cells, which have not been shown before. Treatment with 200 µg/mL SCP-0-1 yielded an apoptotic index of 21.63%, and the proliferation inhibitory rate of the HepG2 cells was approximately 30.13%. These results indicate that the antiproliferative activity of SCP-0-1 is mainly attributable to apoptosis rather than autophagy when the HepG2 cells are treated with 200 µg/mL SCP-0-1, which is in accordance with the morphological analysis and biochemical experiments (Figure 3, Figure 4 and Figure 5).

Cells with seriously impaired deoxyribonucleic acid (DNA) that cannot be repaired properly were normally removed through apoptosis. Hence, DNA damage is considered an indicator of apoptosis [33]. SCP-0-1 potentially induced DNA damage, which was confirmed by cell-cycle analysis. DNA damage was confirmed by Hoechst 33258 staining, which is directly associated with apoptosis [34]. Although SCP-0-1 can induce considerable morphological changes in HepG2 cells, SCP-0-1-induced apoptosis has not been studied. Apoptosis could be triggered by diverse stimuli of the intrinsic or extrinsic pathway. The intrinsic apoptotic pathway concerned mitochondrial apoptotic proteins (cytochrome c (Cyt C), caspase-9, Bcl-2, and Bax), whereas the extrinsic pathway involved signal transduction from caspase-8 and death receptors, which were activated downstream of mitochondrial proapoptotic events [35]. The results show that SCP-0-1 induced apoptosis through the intrinsic pathway (Figure 3 and Figure 4). Studies have reported that mitochondria are concerned in cell apoptosis signalling [36], and ΔΨm loss can induce the opening of the mitochondrial transformable pores and the release of small proteins such as Cyt C from mitochondria [37,38], leading to the formation of a complex comprising apoptosis-activating factor 1 (Apaf-1) and caspase-9. This complex opens the way to proteolytic apoptotis. Translocation of Cyt C is one of the main events of mitochondrial dysfunction and subsequent apoptosis. It is also associated with immediate phosphatidylserine exposure [39]. Considering the aforementioned background, we examined Cyt C translocation and found that SCP-0-1 significantly induced Cyt C release from the mitochondria, leading to caspase-3 activation (Figure 4A).

As shown in Figure 4A, SCP-0-1-mediated caspase-9 and -3 processing and subsequent cleavage of the substrate of caspase-3 (i.e., PARP) suggest that the mitochondrial pathway plays a crucial part in SCP-0-1-induced apoptosis. Furthermore, we investigated the role of the Bcl-2 family members from the mitochondria (antiapoptotic proteins Bcl-2 and Bcl-xL and proapoptotic proteins Bax, Bak, Bcl-xS, Bik, and Bim [40]). SCP-0-1 treatment for 24 h drastically reduced the Bcl-2/Bax ratio in the HepG2 cells concentration-dependently (Figure 4B). The translocation of these proteins damaged mitochondrial functions and induces cells to undergo apoptosis through caspase activation. Because caspase-3 activation is inhibited by members of the inhibitors of apoptosis protein (IAP) family [41] The downregulation of survivin (a member of the IAP family) offered further evidence that SCP-0-1-induced antihepatoma effects were connected with the activation of the caspase cascade (Figure 4A).

Although p53 is a tumour suppressor that promotes apoptosis by upregulating Bax and downregulating Bcl-2 [42], p53 can also induce G2/M arrest [43]. We observed that SCP-0-1 treatment reduced Bcl-2 and increased p53 (Figure 4A). This finding implies that SCP-0-1 induced p53-dependent apoptosis in the HepG2 cells. Therefore, we conclude that p53 determines the fate of HepG2 cells treated with SCP-0-1 (i.e., whether cells enter cell-cycle arrest or undergo apoptosis).

SCP-0-1 induced apoptosis through intrinsic mitochondrial apoptosis because this homogeneous polysaccharide enhanced the cleavage of caspase-9, caspase-3, and PARP, reduced Bcl-2 levels, and increased Bax levels. However, SCP-0-1 treatment did not induce the activation of caspase-8, which is involved in the extrinsic apoptosis pathway (Figure 4A). Therefore, these findings suggest that SCP-0-1 induces apoptosis through the intrinsic rather than extrinsic one in HepG2 cells.

Cell death can be triggered through various mechanisms such as apoptosis, autophagy, necrosis, or a complex of these processes [44]. Autophagy is an ancient cell survival pathway that prevents bioenergetic failure when cells experience metabolic stress [45]. The induction of autophagy is a promising new target in cancer treatment. Cancer cells experience stress when exposed to therapeutic agents, which elicit an autophagic response from such cells. Under certain conditions, autophagy has been considered to be a type of cell death mechanism, for it causes autophagic (type II programmed) cell death [46]. If the condition is too severe or continues for too long, autophagy may even become cytotoxic or result in cell death [47,48]. The increased conversion of LC3A to LC3B and reduced p62 expression, which occur on autophagy induction, are commonly used as markers of autophagy. However, our findings indicate that SCP-0-1 is not a potent inducer of autophagy in HepG2 cells. This conclusion was confirmed by AO staining and Western blotting (Figure 3D and Figure 4C).

The interaction between autophagy, apoptosis, and cell death is not conclusive. A previous study revealed that 2-amino-2-[2-(4-octylphenyl)]-1,3-propanediol hydrochloride (FTY720) can induce the caspase-independent programmed cell death, with autophagy playing a protective part in two cancer cell types [49,50]. Jing et al. [51] demonstrated that hydrochloride (SKF-96365) induced G2/M cell-cycle arrest and apoptosis with accompanying autophagy in colorectal cancer cells, whereas SKF-96365 also induced autophagy, playing a cytoprotective part and delaying apoptosis. However, as in previous studies, restoration of klotho gene expression was shown to induce both autophagy and apoptosis in two cancer cell types, and an autophagy inhibitor to significantly inhibit klotho-induced cell apoptosis [52,53]. Depending on cell type, phase, genetic background, stimuli, and microenvironment, autophagy, apoptosis, and cell death may exert inhibitory, additive, or synergistic effects [54]. Our study shows that SCP-0-1 can induce the apoptosis of the HepG2 cells but shows limited effects on autophagy. Moreover, SCP-0-1 induces mitochondrial apoptosis in the HepG2 cells, which is supported by the aforementioned evidence.

We investigated SCP-0-1-induced apoptosis in HepG2. Our results demonstrate that SCP-0-1 inhibited HepG2 cell growth and proliferation. In addition, we delineated the mechanism underlying the antitumour activity of SCP-0-1 against HepG2 cells.

MAPKs is a family of highly conserved serine/threonine protein kinases evolutionarily affecting many processes of cell-cell survival, apoptosis, proliferation, differentiation, etc. [27]. Additionally, the MAPK signalling pathways have been recently shown to be connected with autophagy. For instance, after being treated with arsenic trioxide (ATO), both apoptosis and autophagy were observed in glioblastoma cells, regulated by the activation of the MAPK signalling pathways [28]. Another study has found that platycodin D (PD) induce autophagy in HepG2 cells. ERK activation may be the key mechanism triggering autophagy. Autophagy inhibition by chloroquine and bafilomycin A1 strengthened PD-induced cytotoxicity and apoptosis [55]. In the current study, SCP-0-1-induced downregulation of the level of Bcl-2 and SCP-0-1-induced apoptosis will not cease by adding an ERK inhibitor (U0126) to the HepG2 cells (Figure 5B). In other words, U0126 cannot rescue SCP-0-1-induced apoptosis.

The PI3K/AKT/mTOR signalling pathway is a crucial intracellular signalling pathway in modulating cell survival, death, proliferation and apoptosis [56]. It is a crucial target in cancer therapy and is negatively associated with both autophagy and apoptosis. Previous studies have proposed AKT’s core position in signalling for cell proliferation and survival, and it is widely considered to be the central target in future anticancer drug development [57]. A study has proved that activated AKT can phosphorylate mouse double minute 2 homolog (MDM2) to accelerate p53 degradation, therefore receding the episodes of p53-mediated apoptosis [58]. Another study has demonstrated that AKT activation can suppress procaspase-9-mediated apoptosis under Bcl-2 family protein control [59].

A previous study has indicated that mTOR is a key point that negatively dominates autophagy, and antineoplastic agents which can inhibit the PI3K/AKT/mTOR axis presumptively irritate autophagy [29]. Our results show that AKT rather than ERK is involved in SCP-0-1-induced apoptosis, in stark contrast to ERK’s dominance represented in other studies [60]. This may be because the cell types and interferences used differ. The members of MAPKs and PI3K/AKT show different effects in regulating apoptosis in accordance with the experimental conditions (Figure 5A).

Hsp90, a molecular chaperone, brings hope in treating cancer and shows necessity in conformational folding and retaining stability of plentiful client proteins including the proper protein folding and intracellular disposition of the diverse proteins involved in cell signalling and survival [61,62]. Among the client proteins (some are oncogenic proteins) altered by Hsp90 chaperones, ranging from transcription factors, kinases to anti-apoptotic molecules, are involved in cancer cell proliferation, cell-cycle, survival, invasion, metastasis, and angiogenesis [30]. Past researches have indicated that Hsp90 inhibition can induce the destabilisation and degeneration of client proteins like AKT and survivin [63]. Hsp90 inhibition can markedly increase the cells ratio in the G2/M phase [64], consistent with the conclusion obtained in this study.

In our study, we found that SCP-0-1 decreases the level of p-AKT and Hsp90 in a concentration-dependent manner. The PI3K inhibitor LY294002 and Hsp90 inhibitor 17-AAG further promote SCP-0-1-induced apoptosis (Figure 5C,D). However, the ERK inhibitor U0126 does not block SCP-0-1-induced apoptosis, suggesting that the Hsp90/AKT signalling pathway is related to SCP-0-1-induced apoptosis (Figure 5B).

## 4. Materials and Methods

### 4.1. Materials

Dried fructus *S. chinensis* (Turz.) Baill was purchased from Beijing Tongrentang (Bozhou, China), produced from Dandong, China, and authenticated by L.H. Wu at the Institute of Chinese Materia Medica, Shanghai University of Traditional Chinese Medicine (Shanghai, China). Diethylaminoethyl cellulose (DEAE)-cellulose, and Superdex 75 and 200 were gained from GE Healthcare Life Sciences (Uppsala, Sweden). Sheep anti-rabbit immunoglobulin Cyt C (1:1000); PARP (1:1000); caspase-3, -8, and -9 (1:1000); Bcl-2 (1:1000); Bax (1:1000); p53 (1:1000); survivin (1:1000); p62 (1:1000); LC3B (1:1000); mitogen-activated protein kinase p38 (p38) (1:1000); ERK; p-ERK (1:1000); c-Jun N-terminal kinase (JNK) and phosphorylated c-Jun N-terminal kinase (p-JNK) (1:1000); AKT and p-AKT (Ser473) (1:1000); Hsp90 (1:1000); cytochrome c oxidase IV (COX IV) (1:1000); and β-actin (1:1000) were purchased from Cell Signaling Technology (Danvers, MA, USA). Secondary polyclonal antibodies were also purchased from Cell Signaling Technology. The molecular weight (*M*_W_) standards were obtained from Thermo Fisher Scientific (Rockford, IL, USA). Inhibitors (U0126, LY294002, and 17-AAG, 3-[4,5-dimethyl-2-thiazolyl]-2,5-diphenyltetrazolium bromide (MTT), Hoechst 33258 and AO were obtained from Sigma (Saint Louis, MO, USA).

### 4.2. Extraction, Separation and Purification of S. chinensis Polysaccharide-0-1

The extraction, separation and purification of the homogeneous polysaccharide SCP-0-1 are illustrated in the flow diagram in Figure 1A [65]. The dried fructus *S. chinensis* (Turz.) Baill were defatted with 95% EtOH for 24 h and dehydrated in air at room temperature. Subsequently, the residues were obtained by boiling with distilled water three times (for 3 h each time). Combined aqueous extracts were concentrated to a suitable volume in a rotary evaporator at 60 °C under reduced pressure and filtered. The filtrate was concentrated and precipitated with 4 volumes of 95% EtOH, left to stand overnight at 4 °C, and centrifuged at 4000 r/min (4 °C, 15 min). The water-soluble extract was dialysed against distilled water for 48 h, then vacuum-dried at −55 °C to gain the raw polysaccharide SCP. The DEAE-cellulose column (50 × 5 cm, Cl^−^ form) was then used to separate the SCP and eluted gradatim with distilled water and 0.1, 0.2, 0.5, and 1.0 mol/L NaCl (Figure 1B). The fraction eluted with distilled water (SCP-0) was further separated on a Superdex 75 column (100 × 2.6 cm), monitored using the highly sensitive Refractive Index Detector RI-102 (Shodex, Tokyo, Japan), and eluted with distilled water to obtain three major fractions: SCP-0-1 (130–140 min), SCP-0-2 (190–205 min), and SCP-0-3 (235–245 min) (Figure 1C). The first fraction, SCP-0-1, was purified on a Superdex 200 column then eluted with distilled water to obtain the homogeneous polysaccharide SCP-0-1 (Figure 1D).

### 4.3. Homogeneity and Molecular Weight

Homogeneity and molecular weight were studied with HPGPC on KS-804 and KS-802 columns (ID: 8 mm, length: 300 mm; Shodex). The columns were precalibrated with pullulan standards, which were used as standard molecular markers because of the known molecular weights (i.e., Pullulan P-5, P-10, P-20, P-50, P-100, P-200, and P-800, Shodex) (Figure S1). The column temperature was 40.0 ± 0.1 °C. The mobile phase was 0.2 mol/L NaCl, and the flow rate was maintained at 0.8 mL/min.

### 4.4. Cell Culture

The hepatoma cell line (HepG2 cells) was bought from the Cell Bank of the Chinese Academy of Sciences (Shanghai, China). HepG2 cells were kept in Dulbecco’s modified Eagle’s medium (DMEM) supplemented with 10% heat-inactivated foetal bovine serum, penicillin (100 units/mL), and streptomycin (100 µg/mL) and incubated at 37 °C in humidified atmosphere of 5% CO_2_.

### 4.5. 3-[4,5-Dimethyl-2-thiazolyl]-2,5-diphenyltetrazolium bromide (MTT) Assay

Cell viability after treatment with SCP-0-1 was assessed by performing the MTT assay in triplicate. The HepG2 cells were incubated at a density of 5 × 10^3^ per well in 96-well microtiter plates. After 24 h, the cells were collected by treatment with trypsin and washed with phosphate buffer saline (PBS), and the medium was substituted with fresh a one. They were then incubated with 20 µL of MTT (5 mg/mL) in each well for 4 h at 37 °C. The absorbance was measured in a microplate-reader (BioTek, Winooski, VT, USA) at an absorbance of 570 nm.

### 4.6. Flow Cytometric Cell-Cycle Analysis

The HepG2 cells were gathered, washed with PBS, and fixed in 70% EtOH for 2 h at 4 °C. Later, they were washed with PBS and resuspended in 0.2 mL of PBS containing 1 mg/mL propidium iodide and 0.1% Triton X-100. Samples were incubated in the darkness for 30 min at 4 °C. Subsequently, the total percentage of apoptotic cells was analysed by Cytomics™ FC500 flow cytometer (Beckman Coulter, Brea, CA, USA).

### 4.7. Annexin-V/Phosphatidylinositol Staining

Annexin-V/PI stainings were studied with the Annexin-V-FITC Apoptosis Detection Kit (Keygen Biotech, Nanjing, China). The HepG2 cells were cultured under hypoxia and then digested with 0.25% trypsin-ethylenediaminetetraacetic acid (EDTA), washed twice by PBS, and centrifuged for 5 min at 3000 r/min. The cells were resuspended in 500 µL of 1 × binding buffer at the concentration of 5 × 10^5^ cells/mL. The cells were stained with 5 µL of Annexin-V-FITC and 5 µL of PI and gently blended and incubated in the dark for 10 min at 37 °C. Subsequently, 400 µL of the cell suspension was transferred to flow tubes. The stained cells were analysed by Cytomics™ FC500 flow cytometer.

### 4.8. Phase Contrast Microscopy

Cell morphological changes were studied using phase contrast microscopy. The HepG2 cells were incubated in 12-well plates and treated with diverse concentrations of SCP-0-1 (0–200 µg/mL) for 24 h. Thereafter, the cells were photographed using on a CCD DP27 inverted phase contrast microscope camera (Olympus, Tokyo, Japan). Objective lens: 40, numeric aperture: 0.65, ocular lens: 10 to get the 400× magnification.

### 4.9. Transmission Electron Microscopy

The HepG2 cells were fixed in PBS with 2.5% glutaraldehyde, pH 7.4, post-fixed in 1% osmium tetroxide, followed by en bloc staining with 2% uranyl acetate. Samples were dehydrated in succession by adding ethanol concentrations and embedded in Epon 812 resin (SPI Supplies, West Chester, PA, USA). Sections were performed using a Tecnai 12 Biotwin TEM (FEI, Eindhoven, The Netherlands).

### 4.10. Hoechst 33258 Staining

After SCP-0-1 treatment, the HepG2 cells were stained with Hoechst 33258 (5 µg/mL) for 30 min at 37 °C and fixed in 4% paraformaldehyde for 10 min. After the cells were washed with PBS three times, the nuclear morphology was observed under the PL2X inverted fluorescence microscope (Olympus). Fluorescence was measured at λex: 350 nm, λem: 460 nm.

### 4.11. Detection of Acidic Vesicular Organelles

The HepG2 cells were incubated into 6-well plates overnight. After SCP-0-1 treatment, the cells were stained with AO (1 mg/mL) for 15 min at 37 °C. After the cells were washed three times with PBS, they were observed promptly under the PL2X inverted fluorescence microscope. Fluorescence was measured at λex: 492 nm, λem: 640 nm.

### 4.12. Western Blotting

The HepG2 cells were washed twice with ice-cold PBS solubilised in 1× lysis buffer (50 mmol/L Tris (pH 6.8), 2% sodium dodecyl sulfate (SDS), and 10% glycerol) with complete protease inhibitors (EDTA-free tablets) (Roche, Basel, Switzerland) and phosphatase inhibitors (Roche) on ice. The cell lysates were subsequently resolved on SDS-polyacrylamide electrophoresis (PAGE) gel and transferred onto nitrocellulose membranes (Millipore, Bedford, MA, USA). After sealing, the membranes were incubated with the specified primary antibodies. The membranes were washed three times in wash buffer then incubated with horseradish peroxidase-conjugated secondary antibodies. Protein bands were detected using a chemiluminescence detection system. The band in the electrophoresis maps were densitometrically analysed using a quantitative gel imaging Quantity One software (Bio-Rad, Hercules, CA, USA).

### 4.13. Cytochrome C Release

The mitochondrial and cytosolic proteins from the treated HepG2 cells were fractionated as instructed by Nencioni et al. [66]. In brief, the cells were gathered and resuspended in 100 µL of lysis buffer containing 0.025% digitonin, 250 mmol/L sucrose, 20 mmol/L 4-(2-hydroxyethyl)-1-piperazineethanesulfonic acid (hepes free acid) (pH 7.4), 5 mmol/L MgCl_2_, 10 mmol/L KCl, 1 mmol/L EDTA, 1 mmol/L phenylmethanesulfonyl fluoride, 10 µg/mL aprotinin, and 10 µg/mL leupeptin. After being incubated at 4 °C for 10 min, the cells were centrifuged (2 min at 13,000× *g*), and the supernatant (cytosolic fraction) was deserted. The remaining digitonin-insoluble pellet was dissolved in 2% SDS buffer to obtain a membrane-bound organellar fraction enriched with mitochondria. Equal amounts of proteins (50 µg/lane) from these two fractions were decomposed on 10% SDS-PAGE gel. Proteins were transferred onto polyvinylidene fluoride membranes and analysed via using immunoblotting with the anti-Cyt C antibody.

### 4.14. Statistical Analyses

The data were shown as mean values ± SD and were calculated by using the Student *t* test and analysis of variance in accordance with the number of groups compared. Statistical analyses were performed for data collected from at least three independent experiments. Differences with 95% confidence (*p* < 0.05) were considered significant (*). The data were analysed using PASW Statistics version 18.0 (SPSS Inc., Chicago, IL, USA).

## 5. Conclusions

**Figure 6:**
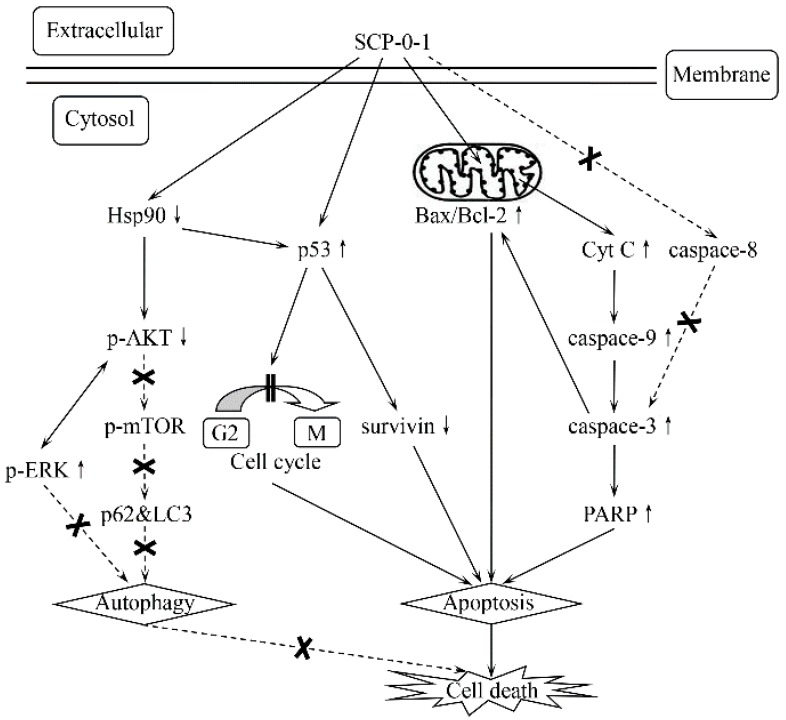
*S. Chinensis* polysaccharide-0-1 (SCP-0-1) induced molecular events of mitochondrial apoptosis rather than autophagy. Up-arrows and down-arrows denote the action of positive and negative regulation of specific proteins, respectively. Olid line arrows indicates pathways direction. Crosses and dash line arrows are represented as no appreciable effects on specific pathways. Double vertical lines refers to the induction of cell cycle G2/M arrest.

In our study, we investigated the antiproliferative activity of SCP-0-1 and its action mechanisms in HepG2 cells (Figure 6). Our preliminary experiments showed that a novel polysaccharide, SCP-0-1, inhibited the growth of HepG2 cells dose-dependently (0–200 µM). The growth inhibition induced by SCP-0-1 was connected with a growth in the subG0/G1 apoptotic population of the HepG2 cells. SCP-0-1 increased the number of apoptotic cells dose-dependently, as assessed using FCM. Our study demonstrated that SCP-0-1 exerts its antiproliferative activity and induces mitochondrial apoptosis rather than autophagy by inhibiting the Hsp90/AKT signalling pathway in an ERK-independent manner.

The efficacy of SCP-0-1 in inhibiting the growth of HepG2 cells in vitro suggests its potential application in hepatocellular carcinoma (HCC) therapy. Therefore, the present data on SCP-0-1 can promisingly increase the interest and understanding of its functions among cancer scientists and evoke further preclinical and clinical research on *S. chinensis* polysaccharides.

## Supplementary Materials

Supplementary materials can be found at http://www.mdpi.com/1422-0067/17/7/1015/s1.
