# Supplementary material for: A Homogeneous Polysaccharide from Fructus Schisandra chinensis (Turz.) Baill Induces Mitochondrial Apoptosis through the Hsp90/AKT Signalling Pathway in HepG2 Cells
Source: Int J Mol Sci. 2016 Jun 28;17(7):1015. doi: 10.3390/ijms17071015 (PMC4964391; doi:10.3390/ijms17071015)
Supplement: Supplementary file 1 [file ijms-17-01015-s001.pdf]

Supplementary Materials: A Homogeneous Polysaccharide from Fructus *Schisandra chinensis* (Turz.) Baill Induces Mitochondrial Apoptosis through the Hsp90/AKT Signalling Pathway in HepG2 Cells

Yonglin Chen, Songshan Shi, Huijun Wang, Ning Li, Juan Su, Guixin Chou and Shunchun Wang

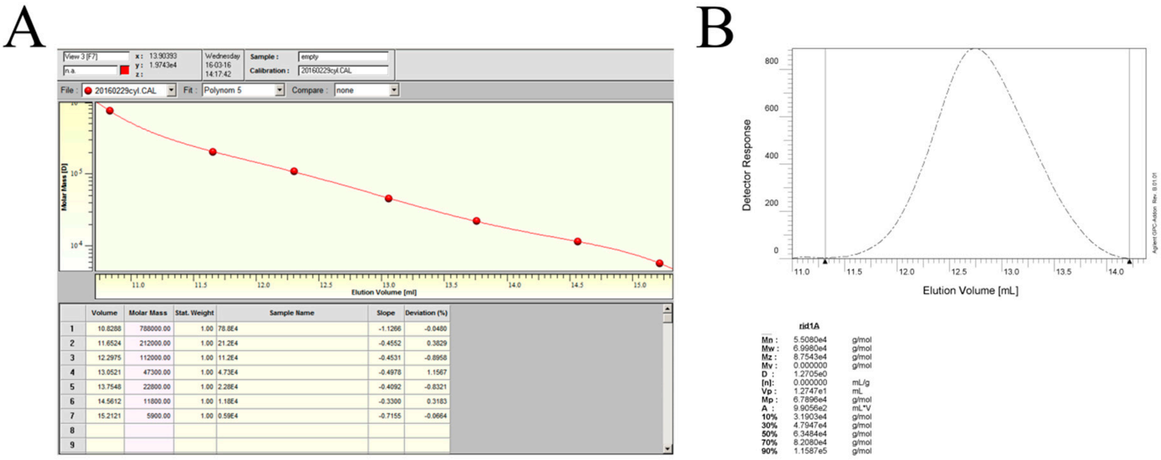

Figure S1. The molecular weight of *S. chinensis* polysaccharide-0-1. (**A**) Standard molecular markersin reference to P-series Dextran (*i.e.*, Pullulan P-5, P-10, P-20, P-50, P-100, P-200, and P-800, Shodex); (**B**) The molecular weight of *S. chinensis* polysaccharide-0-1 (SCP-0-1) was estimated to be 69.980 kDa.

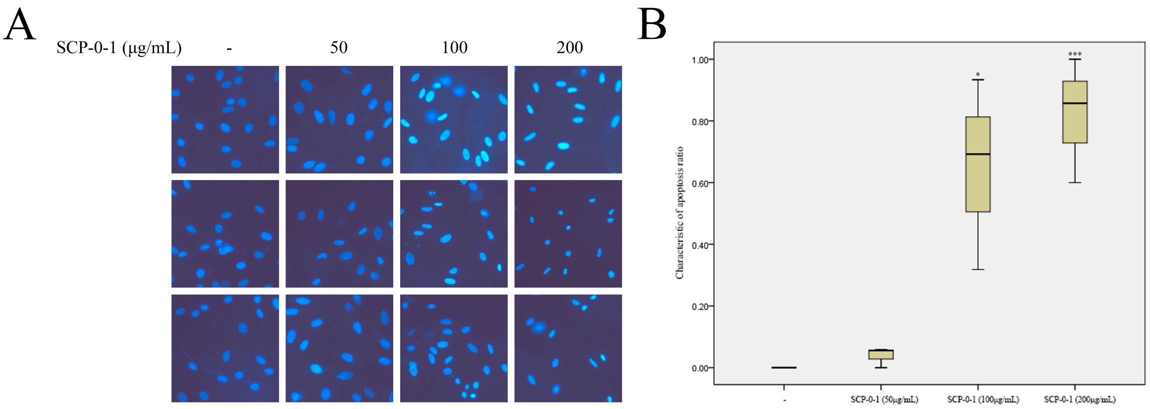

Figure S2. Nuclear morphology staining using Hoechst 33258. (**A**) The morphological characterisation of cell nuclei was analysed under a fluorescence microscope (magnification: 400×). Condensed chromatin are shown to contain compacted chromatin, which is characteristic of apoptosis; (**B**) Its related data (characteristic of apoptosis ratio) were presented as the mean values ± SD of three independent experiments. * $p < 0.05$ and *** $p < 0.001$ versus control group.

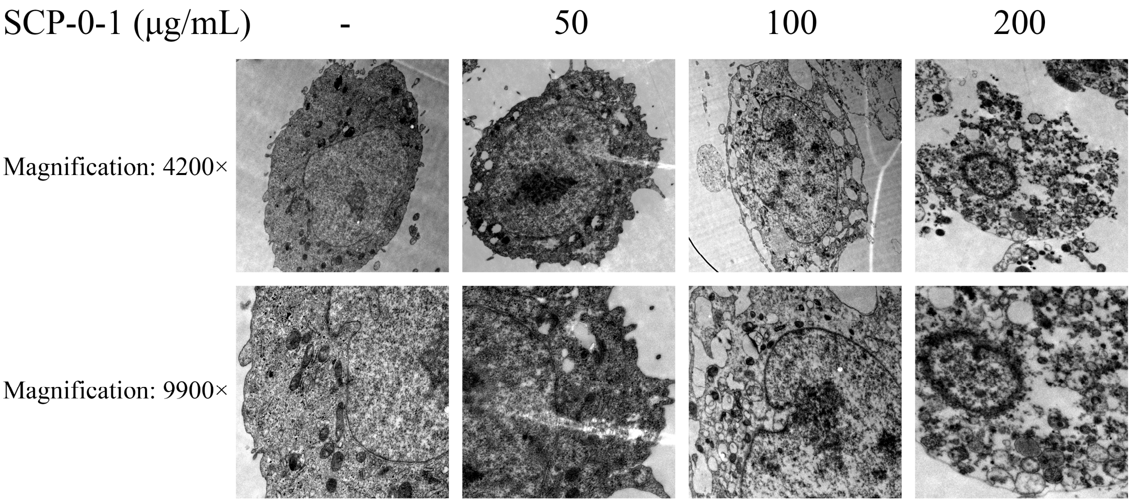

Figure S3. Morphological changes induced by *S. chinensis* polysaccharide-0-1. After treatment with 100–200 μg/mL SCP-0-1 for 24 h, cellular morphology was observed under a transmission electron microscope (magnification: 4200× and 9900×). An inhibitory effect of SCP-0-1 on cell growth is observed as being accompanied by membrane blebbing and condensed chromatin, which are characteristic of apoptosis.
